# An observational study of health insured visits for children following Medicaid eligibility expansion for adults among a linked cohort of parents and children

**DOI:** 10.1097/MD.0000000000030809

**Published:** 2022-09-23

**Authors:** Heather Angier, Tahlia Hodes, Laura Moreno, Jean O’Malley, Miguel Marino, Jennifer E. DeVoe

**Affiliations:** a Oregon Health & Science University, Portland, OR, USA; b Fred Hutchinson Cancer Center, Seattle, WA, USA; c OCHIN, Inc., Portland, OR, USA.

**Keywords:** Affordable Care Act, child health, family health, health insurance

## Abstract

Despite its focus on adults, the Affordable Care Act (ACA) Medicaid expansion led to increased health insurance enrollment for children in the United States. Previous studies looked at parent and child insurance changes separately, or used a single survey response item to understand changes in health insurance for parents and children. It is, however, important to understand the connection between parent and child insurance changes together (not individually) using data sources that account for insurance over time. Therefore, to understand the association of parental health insurance on their children’s coverage, leveraging a cohort of linked families seen in community health centers (CHCs), we used electronic health records to link a cohort of parents and children with ≥1 visit to a CHC in a Medicaid expansion state pre- (1/1/2012–12/31/2013) and ≥1 visit post-ACA (1/1/2014–12/31/2018) and determined primary payer type for all visits. This observational, cohort study assessed the rate of insured visits for children pre- to post-ACA across four parental insurance groups (always insured, gained Medicaid, discontinuously insured, never insured) using Poisson mixed effects models. We included 335 CHCs across 7 United States. Insurance rates were highest (~95 insured visits/100 visits) for children of parents who were always insured; rates were lowest for children of parents who were never insured (~83 insured visits/100 visits). Children with a parent who gained Medicaid had 4.4% more insured visits post- compared to pre-ACA (adjusted relative rates  = 1.044, 95% confidence interval: 1.014, 1.074). When comparing changes from pre- to post-ACA between parent insurance groups, children’s insured visit rates were significantly higher for children of parents who gained Medicaid (reference) compared to children of parents who were always insured (adjusted ratio of rate ratio: 0.963, confidence interval: 0.935–0.992). Despite differences in Medicaid eligibility for children and adults, health insurance patterns were similar for linked families seen in CHCs. Findings suggest consideration should be paid to parent health insurance options when trying to increase children’s coverage.

## 1. Introduction

Health insurance coverage is associated with improved access to healthcare services, and fewer unmet healthcare needs.^[1]^ Despite the 1997 Children’s Health Insurance Program (CHIP) and its 2009 Reauthorization, some eligible children remain uninsured.^[2,3]^ The Affordable Care Act (ACA) expanded Medicaid eligibility to ≤138% of the federal poverty level (FPL) for adult United States (US) citizens or permanent residents in states that adopted the provision,^[4]^ which led to 11 million new adult Medicaid enrollees,^[5]^ many of whom were parents. Despite its focus on adults, the ACA Medicaid expansion also led to increased health insurance enrollment for children.^[6–8]^ It has been theorized that these increases in child health insurance rates were due to outreach, coverage mandates, and simplified application and eligibility processes.^[6,7]^ There may be other unknown mechanisms as well.

Previous studies looked at parent and child health insurance changes separately,^[8]^ or used a single survey response item to understand changes in health insurance for parents and children.^[6,7]^ It is important; however, to understand parent and child insurance changes together (not individually) as families likely influence one another. Survey data rely on one question to determine health insurance coverage,^[6,7]^ whereas electronic health record data (EHR) include actual payer information for a healthcare visit over time. Surveys also tend to overstate health insurance coverage.^[9]^ Therefore, we used healthcare visit-based information to assess the association between health insurance change for linked parents and children who both receive healthcare together in a network of community health centers (CHCs).

The network of CHCs has a single EHR instance. The EHR includes health insurance tied to healthcare receipt, not self-reported coverage. As CHCs see patients regardless of insurance status, they cared for many uninsured adults prior to the ACA Medicaid expansion and continued to care for those who remained uninsured afterwards. CHCs also predominantly care for families with low-incomes; 91% of CHC patients are in or near poverty; therefore, children seen in CHCs are likely eligible for CHIP coverage.^[10]^ In addition, the majority of CHCs were providing enabling services to patients including Medicaid eligibility assistance prior to 2014 ACA health insurance outreach efforts.^[11]^ Thus, children’s coverage in this setting should not be impacted by parental insurance because the majority of children seen in CHCs are eligible for CHIP coverage based on income and the clinics had resources to help children gain coverage. Yet, we hypothesized that children receiving care in CHCs would have a higher likelihood of insured visits after their parents gained Medicaid post-ACA than children of parents who did not. We thought parent coverage would still impact these children based on previous research highlighting the relationship between parent and child coverage before the ACA Medicaid expansion.^[12–15]^

## 2. Methods

For this observational study, we leveraged a cohort of linked parents and children using EHR data from the OCHIN (not an acronym) network of CHCs, described elsewhere.^[16]^ Briefly, we identified children with a visit and used both the emergency contact and guarantor fields to find linked parents who also received healthcare in a networked CHC. After we established the cohort, we used data from the Accelerating Data Value Across a National Community Health Center clinical research network of PCORnet^®^.^[17]^ We selected parents with ≥1 visit pre- (1/1/2012–12/31/2013) and ≥1 visit post-ACA (1/1/2014–12/31/2018). We included children who were younger than 18 years of age at their first visit after 1/1/2012 with ≥1 visit pre- and ≥1 post-ACA across 7 US states that expanded Medicaid on 1/1/2014 (California, Massachusetts, Minnesota, Nevada, Ohio, Oregon, and Washington). Accelerating Data Value Across a National Community Health Center data are routinely assessed for completeness and quality and have low missingness on relevant variables including insurance status.^[18]^

Parent insurance was categorized into four mutually exclusive groups: *always insured* = at least one parent was always insured, *gained Medicaid* = at least one parent was uninsured at all visits pre-ACA visits post-ACA were paid for by Medicaid; *discontinuously insured* = at least one parent had visits both pre-and post-ACA included some insured and some uninsured visits; and *never insured* = at least one parent was never insured. If a child had two parents with different insurance patterns, we chose the most insured parent’s status due to previous research on the relationship between parent and child coverage before the ACA Medicaid expansion.^[12–15]^ Insurance status for both child and parent was determined by primary payer type for visits.

The outcome was insured child visit rates to the CHC. To model this outcome, we compared the change in rate of insured child (publicly or privately) visits from pre- to post-ACA by parent insurance group.

The following EHR-derived child covariates were hypothesized to as potential confounders and considered in analyses: age at first visit, sex, federal poverty level at first visit (≤138%, >138%, unknown), number of chronic conditions on the problem list throughout the study period, race/ethnicity (non-Hispanic white, non-Hispanic Black, Hispanic, other), and state (Oregon, California, other). States were combined into other due to low sample size.

### 2.1. Statistical analysis

First, we described our population by examining characteristics of our mothers, fathers, and children in our study sample. To model the change in rate of insured child visits from pre- to post-ACA, we used a Poisson mixed effects model to estimate adjusted relative rates (aRRs). We included a random intercept for children to account for clustering of temporal observations within a child. We utilized this Poisson model to estimate the covariate-adjusted rate of child insured visits for the pre- and post-ACA expansion time periods by parent’s insurance group. To determine if there was a differential effect in change of insurance rates from pre- to post-ACA between parental insurance groups, we evaluated a two-way interaction term between expansion period (pre to post) and insurance group (always insured, gained Medicaid, discontinuously insured, never insured). The reference was the gained Medicaid group. We used SAS software (SAS Institute Inc., Cary, NC) version 9.4. Statistical significance was set at Type 1 error of 0.05.

The study was reviewed and approved by the Oregon Health & Science University’s Institutional Review Board. Consent and authorization were waived for this minimal risk study of secondary use of EHR data.

## 3. Results

The analysis included 28,104 mothers, 4266 fathers, and 31,524 children from 335 CHCs. The mean age of the children in 2014 was 7 years, 52% were Hispanic, 77% had a family income ≤138% FPL, 64% preferred speaking English and they were evenly split in terms of sex. Most of the children had at least one parent who was discontinuously (51%) or always (39%) insured, whereas 7.8% had a parent who was never insured, and 2.4% had a parent who gained Medicaid. The mean age of mothers in 2014 was 34, whereas the mean age for fathers was 42. 52% of mothers and 34% of fathers were Hispanic. Mothers had an average of nearly 5 visits per year and fathers had 3. Insurance status for parents differed for mothers and fathers; 13% of fathers and 7% of mothers were never insured, 37% of fathers and 53% of mothers were discontinuously insured, and 45% of fathers and 38% of mothers were always insured (see Table 1).

**Table 1:** Characteristics of mothers, fathers, and children in the study population.

		Mothers	Fathers	Children
N		28,104	4266	31,524
Age in 2014, mean (SD)		33.61 (8.29)	41.79 (8.98)	7.20 (5.22)
Age at first visit, mean (SD)		30.46 (8.39)	39.03 (8.96)	4.72 (4.89)
Race/ethnicity, N (%)	Hispanic	14,709 (52.3)	1468 (34.4)	16,284 (51.7)
	Non-Hispanic Black	1941 (6.9)	211 (4.9)	2176 (6.9)
	Non-Hispanic White	9487 (33.8)	1980 (46.4)	10,526 (33.4)
	Other	1967 (7.0)	607 (14.2)	2538 (8.1)
FPL at first visit, N (%)	≤138%	22,156 (78.8)	2894 (67.8)	24,386 (77.4)
	>138%	3778 (13.4)	719 (16.9)	4369 (13.9)
	Unknown	2170 (7.7)	653 (15.3)	2769 (8.8)
Visits per year, mean (SD)		4.84 (3.45)	3.28 (3.26)	3.00 (1.98)
Number of chronic conditions, mean (SD)		2.70 (2.62)	2.96 (2.76)	1.06 (1.33)
State, N (%)	CA	9427 (0.34)	1109 (0.26)	10,379 (32.9)
	OR	15,749 (0.56)	2849 (0.67)	17,950 (56.9)
	Other	2928 (0.10)	308 (0.07)	3195 (10.1)
Sex, N (%)	Female	28,104 (100)	0 (0)	15,710 (49.8)
	Male	0 (0)	4266 (100)	15,814 (50.2)
English preferring, N (%)	Yes	15,556 (0.55)	2515 (0.59)	20,148 (63.9)
	No	12,548 (0.45)	1751 (0.41)	11,376 (36.1)
Insurance category of parent, N (%)*	Always	10,556 (37.6)	1913 (44.8)	12,241 (38.8)
	Never	2071 (7.4)	573 (13.4)	2459 (7.8)
	Gained	585 (2.1)	183 (4.3)	751 (2.4)
	Discontinuous	14,892 (53.0)	1597 (37.4)	16,073 (51.0)

CA = California, FPL = federal poverty level, OR = Oregon, SD = standard deviation.

* At least one parent with insurance type.

**Figure 1.**
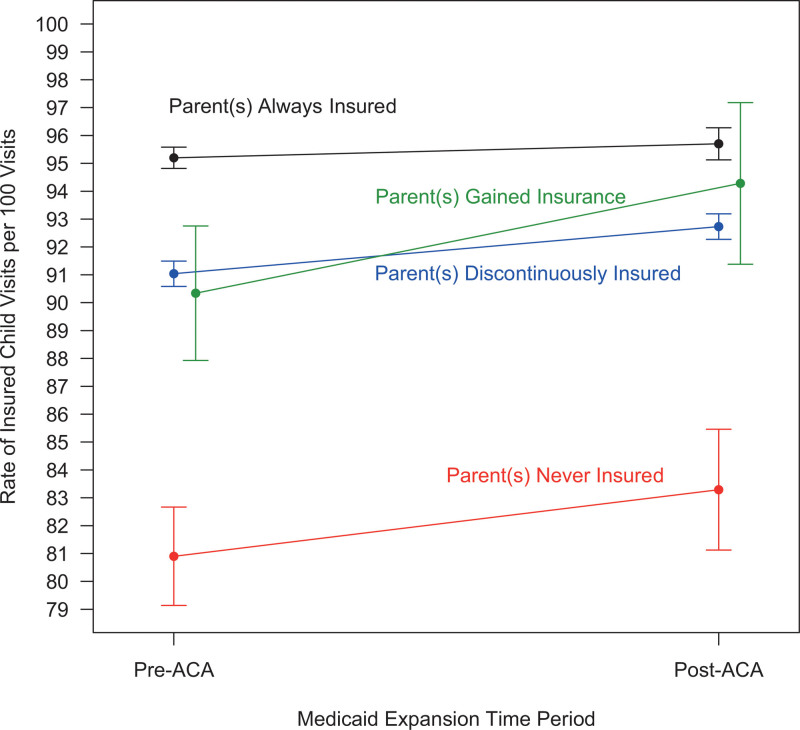
Adjusted rates of child insured visits pre- to post-ACA by parent health insurance group. The numerator was the number of insured visits and the total number of visits served as the denominator. Parent insurance categories: *always insured* = at least one parent was always insured, *gained Medicaid* = at least one parent was uninsured at all visits pre-ACA visits post-ACA were paid for by Medicaid; *discontinuously insured* = at least one parent had visits both pre-and post-ACA included some insured and some uninsured visits; and *never insured* = at least one parent was never insured. Adjusted for the following child covariates: age at first visit, sex, federal poverty level at first visit (≤138%, >138%, unknown), number of chronic conditions on the problem list throughout the study period, race/ethnicity (non-Hispanic white, non-Hispanic Black, Hispanic, other), and state (Oregon, California, other). States were combined into other due to low sample size. ACA = Affordable Care Act.

Adjusted results suggest insurance rates were highest for children of parents who were always insured (both pre- and post ACA, ~95 insured visits per 100 visits) and children of parents who were never insured had the lowest rates (pre-ACA: ~81 insured visits per 100 visits, post-ACA: ~83 insured visits per 100 visits) (see Fig. 1).

Children with at least one parent who gained Medicaid had 4.4% more insured visits post- compared to pre-ACA (aRR = 1.044, 95% confidence interval [CI]: 1.014, 1.074). Children with at least one discontinuously insured parent had 1.9% more insured visits post- than pre-ACA (aRR = 1.019, 95% CI: 1.014, 1.023).

When comparing changes from pre- to post-ACA between parent insurance groups, analyses showed children’s insured visit rates were significantly higher for children of parents who gained Medicaid (the reference group) compared to the children whose parents were always insured [adjusted ratio of rate ratio: 0.963, CI: 0.935–0.992] (see Table 2).

**Table 2:** Within and between-group changes in child health insured visits pre- to post-ACA by parental health insurance category.

	Within group comparison	Between group comparison
Parent(s) insurance coverage pattern from pre- to post-ACA	Relative rate change from pre- to post ACA (95% CI)	Relative differences between parent(s) insurance coverage groups in rate changes from pre- to post-ACA (95% CI)
Always insured	**1.005 (1.001, 1.009**)	**0.963 (0.935, 0.992**)
Gained Medicaid*	**1.044 (1.014, 1.074**)	Reference
Discontinuously insured	**1.019 (1.014, 1.023**)	0.976 (0.948, 1.005)
Never insured	**1.030 (1.008, 1.051**)	0.987 (0.952, 1.023)

ACA = Affordable Care Act, CI = confidence interval Bold is statistically significant at <.05.

* The reference for between group comparisons was parent gained Medicaid group.

## 4. Discussion

In this sample of linked children and parents receiving healthcare together in CHCs, children’s rates of insured visits followed similar patterns to their parents’ insurance group. For example, children with an always insured parent had very high insured visit rates both pre- and post-ACA (~95 insured visit per 100 visits). Children with parents who were never insured had between 80 and 83 insured visits per 100 visits. We do not know why 20% of the visits for children with parents who were never insured remained uninsured post-ACA. Medicaid and more research is needed to understand this group. One reason could be that some of the children are not US citizens or legal residents (or their parents are not) and therefore have limited access to coverage.^[19]^ California began to allow children to enroll in Medicaid and CHIP regardless of their immigration status in 2016^[20]^ and Oregon started in 2018.^[21]^ Research on the California policy change found an association with a 12 percentage point increase in public health insurance coverage for children from 2016 to 2018.^[22]^ However, our results suggest that expanding eligibility to undocumented children only is not sufficient; parents may need to be covered as well.

Children with at least one parent who gained Medicaid had 4.4% more insured visits post- compared to pre-ACA. This is in alignment with previous research that found increased parental health insurance led to increased child health insurance.^[6,12–15]^ For example, a previous study of the ACA Medicaid expansion found children of parents newly eligible for Medicaid experienced a 5.4% percentage point increase in coverage, despite the child’s longstanding eligibility for insurance prior to parents gaining coverage. This previous study also highlighted that all children experienced a gain in coverage post-ACA despite their parent’s Medicaid eligibility status.^[6]^ Similarly, all children in our study experienced an increase in insured visit rates post-ACA. Further, we found that insured visit rates increased most for children whose parents gained Medicaid, while the rate of increase for children with parents already insured was lower. This finding suggests that despite ACA Medicaid expansion policies primarily targeting adults, children were positively impacted.

### 4.1. Limitations

This analysis provides information about the association between parent and child health insurance, not causation. Our sample consisted of children and parents who could be linked through EHR records and both received care in the same CHC network. The sample is therefore biased toward families that are seen together in the CHC network and those with visits, which may limit generalizability. Sample size constrained us from considering all possible insurance patterns of two parents and instead chose the parent with the most coverage, as we believed this parent’s health insurance would have the most impact on the child. It is also possible that children had different healthcare needs across parent group, yet the mean health condition for the children was 1.06 with a standard deviation of 1.33. We assessed insured visits and did not take into account coverage eligibility. That said, the majority of mothers, fathers, and children in our sample had household incomes ≤138% FPL making them potentially eligible for Medicaid.

## 5. Conclusion

Despite differences in Medicaid eligibility for children and adults, health insurance patterns were similar for linked families seen in CHCs. Findings suggest consideration should be paid to parent health insurance options when trying to increase children’s coverage.

## Author contributions

**Conceptualization:** Heather Angier, Jean O’Malley, Miguel Marino, Jennifer E. DeVoe.

**Data curation:** Jean O’Malley.

**Formal analysis:** Tahlia Hodes.

**Funding acquisition:** Jennifer E. DeVoe.

**Investigation:** Heather Angier.

**Project administration:** Heather Angier.

**Resources:** Heather Angier.

**Supervision:** Heather Angier, Miguel Marino.

**Validation:** Miguel Marino.

**Visualization:** Tahlia Hodes.

**Writing – original draft:** Heather Angier.

**Writing – review & editing:** Heather Angier, Tahlia Hodes, Laura Moreno, Jean O’Malley, Miguel Marino, Jennifer E. DeVoe.
